# Awareness, knowledge, and acceptance of teledentistry among dental practitioners: a cross-sectional survey

**DOI:** 10.1186/s12903-026-07931-5

**Published:** 2026-02-24

**Authors:** Jaana-Sophia Kern, Jennifer Paulssen, Katharina Marx-Schütt, Marta Rizk, Nikolaus Marx, Anna Bock, Isabel Knaup, Stefan Wolfart

**Affiliations:** 1 Department of Prosthodontics and Biomaterials, Centre for Implantology, Uniklinik RWTH Aachen, Pauwelsstr. 30, Aachen, 52074 Germany; 2 Department of Internal Medicine I, Uniklinik RWTH Aachen, Pauwelsstr. 30, Aachen, 52074 Germany; 3 Department of Orthodontics and Orofacial Orthopedics, Uniklinik RWTH Aachen, Pauwelsstr. 30, Aachen, 52074 Germany; 4 Department of Oral and Maxillofacial Surgery, Uniklinik RWTH Aachen, Pauwelsstr. 30, Aachen, 52074 Germany

**Keywords:** Telemedicine, Teledentistry, Referral and consultation, Surveys and questionnaires, Digital health, Store-and-Forward teleconsultation, Dentists

## Abstract

**Background:**

Teledentistry has the potential to enhance access to dental care and interprofessional communication. However, its implementation depends strongly on practitioners’ acceptance and understanding. Therefore, this study aimed to assess dentists’ awareness, knowledge, and acceptance of teledentistry and to examine how professional experience, anticipated frequency of use, and practice size influence their attitudes. Furthermore, the interest in a dedicated teledentistry centre was evaluated.

**Methods:**

A cross-sectional survey was conducted among 362 dentists. A 16-item questionnaire was distributed via SoSci Survey and in paper form. Statistical analysis included chi-square and Fisher-Freeman-Halton exact tests (*P* < .05).

**Results:**

Of the 362 dentists invited, 187 completed the survey, yielding a response rate of 52%. More than 70% of participants were familiar with the term teledentistry, yet only one quarter demonstrated a comprehensive understanding of its scope. About one third (32%) had previously used teledentistry in clinical practice. A large majority (71%) considered the establishment of a regional teledentistry centre beneficial, for improving communication with specialists and managing referrals, although their expectations regarding acceptable response times varied considerably between one day and one week. Interest in teledentistry showed no significant association with years of professional experience (*P* = .76) or the number of dentists in a practice (*P* = .598) but correlated significantly with the frequency of relevant clinical cases (*P* < .01, Cramér’s V = 0.449). Main concerns included diagnostic limitations without in-person contact and data privacy.

**Conclusions:**

The study highlights a generally positive attitude toward teledentistry. The findings provide substantial support for the proposed initiative to establish a teledentistry centre, operating on the premise of the store-and-forward method. Familiarity with the concept and clinical demand influence acceptance more than professional experience or the number of practicing dentists in a dental office.

**Supplementary Information:**

The online version contains supplementary material available at 10.1186/s12903-026-07931-5.

## Background

The COVID-19 pandemic has significantly accelerated the use of digital tools in healthcare [1]. While dental diagnostics and treatment traditionally rely on direct patient contact, recent years have demonstrated the potential of digital technologies to support remote dental care in selected scenarios [2]. Teledentistry, first introduced in the early 1990s through the U.S. military’s “Total Dental Access Project,” was originally designed to provide remote dental support to soldiers via standard telephone lines [3]. Since then, it has evolved substantially and is now regarded as a subdomain of telemedicine, comprising two main delivery modes: asynchronous and synchronous [4–7].

In asynchronous teledentistry (often referred to as the “store-and-forward” model), clinical information such as images, radiographs, and patient histories is collected and transmitted to a consulting specialist, who evaluates the data and provides a diagnosis or treatment plan at a later time [4, 5]. This model enables flexible workflows and minimizes the need for real-time coordination, particularly in routine consultations and case triage as demonstrated in recent studies comparing mobile application-based teledentistry with conventional face-to-face oral examinations, which reported high agreement between remote and clinical assessments [8]. In contrast, synchronous teledentistry involves real-time communication between the dentist and the patient or another provider. This is typically achieved via live video consultations or remote monitoring systems, enabling direct interaction, immediate feedback, and real-time diagnostics [6, 7].

Despite the growing implementation of these technologies, there is limited standardized data on patients’ awareness of and attitudes toward teledentistry [9, 10]. However, the available studies point to positive patient experiences [10]. A comparative study on temporomandibular disorder consultations showed similar satisfaction rates between conventional and teledentistry appointments. Patients highlighted reduced travel needs, time savings, and increased privacy as key benefits [11]. An Australian pilot study involving 489 patients reported that nearly 90% of participants felt their needs were met, their questions were answered, and they were well cared for during teledentistry consultations [12]. These findings suggest high acceptance and the potential to improve access and convenience, particularly in underserved populations. In addition to improving patient satisfaction, teledentistry has also been associated with environmental benefits. A retrospective cross-sectional study assessing pediatric teledentistry visits found that significant travel-related resource savings were achieved - equivalent to 553 gallons of gasoline and 4,875 kg of CO₂ emissions - by avoiding in-person visits [13].

While the majority of dental care continues to rely on hands-on procedures, a substantial body of evidence shows that teledentistry can effectively support key clinical tasks such as diagnosis, monitoring, and treatment planning [6, 14]. Technological advancements have also enabled mobile applications that use smartphone cameras to support remote diagnostic tasks, with particularly promising results reported for the detection of dental caries across diverse patient groups [5, 15–18]. A recent systematic review found high diagnostic accuracy and strong agreement between remote and in-person assessments, concluding that teledentistry contributes to faster diagnoses and improved patient outcomes [19]. Similarly, Gurgel Juarez et al. reviewed multiple systematic reviews and confirmed that teledentistry offers clinical value, particularly in situations where direct contact is limited [20]. For example, patients with special health care needs in long-term care could benefit from teledental services and thus have easier access to dental care [21].

Despite its potential, widespread adoption of teledentistry in Germany has been hampered by several barriers, including insufficient infrastructure, lack of reimbursement structures, and scepticism among dental professionals regarding cost and administrative workload [22, 23]. In a 2021 analysis, Huettig and Schwendicke called for greater research collaboration and infrastructure development to enable broader use of teledentistry [24]. The insights gained from this could be useful both nationally and internationally to expand the necessary teledental infrastructures and services [24]. The few available international studies suggest that dentists are generally open to teledentistry use, provided adequate support and clear frameworks are in place [25–27]. For example, in a national survey of Saudi dentists, half of participants reported having applied teledentistry in practice and the majority perceived it as beneficial [28]. Similarly, a large cross-sectional survey among Indonesian dentists demonstrated generally positive attitudes toward teledentistry, while simultaneously highlighting ongoing concerns related to technical compatibility, data security, and professional responsibility, further underscoring the heterogeneity of dentists’ perceptions across different healthcare settings. Overall, there appears to be substantial heterogeneity in dentists’ awareness, knowledge, and acceptance of teledentistry, with persistent concerns regarding diagnostic responsibility, data protection, and integration into daily workflows [4, 28–30].

Also in the German context, evidence on dentists’ awareness, knowledge, and acceptance of asynchronous (store-and-forward) systems remains scarce. This gap may be partly explained by persistent structural and regulatory barriers, as described by Wolf et al. [23], including limited digital interoperability, incomplete integration of dental practices into secure telemedical networks, and ongoing uncertainties regarding data-protection requirements. These factors appear to hinder the wider adoption of digital communication tools in everyday dental care and underscore the need for empirical data on how dentists currently perceive and evaluate teledentistry. At the University Hospital Aachen, the TELnet@NRW^1^ project has successfully implemented telemedicine structures in emergency and intensive care, accompanied by the creation of an innovation centre for digital medicine [31, 32]. Dentistry, however, has yet to be integrated into this digital infrastructure. Against this background, there is a clear need for empirical data on how dentists perceive, understand, and evaluate teledentistry services in routine practice, particularly with regard to asynchronous store-and-forward applications. Beyond infrastructural considerations, factors such as professional experience, anticipated clinical relevance, and practice context may play an important role in shaping acceptance and intended use among dental practitioners.

Therefore, this study aimed to assess dentists’ awareness, perceived relevance, and acceptance of teledentistry services, with a particular focus on the store-and-forward concept. In addition, we examined how professional experience, anticipated frequency of use, and practice characteristics are associated with interest in teledentistry. In this context, the following three hypotheses were tested.

### Hypothesis 1

H₀: Dentists’ interest in store- and forward teledentistry services is independent of their years of professional experience.

H₁: Dentists with fewer years of professional experience show greater interest in store-and-forward teledentistry services.

### Hypothesis 2

H₀: Dentists’ interest in store-and-forward teledentistry services is independent of the number of clinically relevant cases in which teledentistry could be applied.

H₁: Dentists’ personal interest in store-and forward teledentistry services is associated with the anticipated frequency of their use.

### Hypothesis 3

H₀: The use of store-and forward teledentistry services is independent of the number of dentists working in a dental practice.

H₁: The greater the number of dentists working in a practice, the lower the likelihood of utilising store-and forward teledentistry services.

## Methods

### Study design

#### Target group and sampling

The study population comprised all 575 dentists registered with the local dental chamber in the Aachen region, a moderately populated urban-rural area in the west of Germany. Using publicly available databases and professional directories, we identified 362 eligible practitioners with accessible contact information. The study employed a total population sampling approach rather than random sampling to ensure maximum coverage within this defined region.

*Inclusion criteria*: licensed dentists or dental residents practicing in the “Städteregion Aachen” during the data-collection period (May–December 2023)

*Exclusion criteria*: retired practitioners, dental students, and those without clinical activity. Participation was voluntary and anonymous.

### Survey structure and questionnaire design

The questionnaire was developed based on previous validated surveys assessing knowledge, attitudes, and practices regarding telemedicine and teledentistry [30, 33]. Items were adapted to the local context and reviewed by an expert panel consisting of qualified dentists from Aachen University Hospital. The team of authors was advised by an experienced medical psychologist.

A pre-test among ten dentists at the University Hospital Aachen confirmed clarity and completion time (~ 5 min). During pre-testing, the internal consistency and clarity of the scaled items were reviewed to ensure conceptual alignment and reliability. Minor wording adjustments were made before dissemination.

The questionnaire was designed using SoSci Survey and distributed both electronically and in paper form. It comprised 16 items in total, including 13 closed-ended and 3 open-ended questions, with an average completion time of approximately five minutes. All questions were formulated clearly and neutrally, avoiding double negatives or leading wording. Key terms such as “store-and-forward” were briefly explained to ensure consistent understanding among respondents.

### Questionnaire content

The questionnaire comprised four main sections. The first section covered demographic information, including professional background, years of experience, and the number of dentists working in each practice. The second section focused on practice infrastructure, addressing aspects such as radiographic imaging systems, internet quality, and the overall level of digitalisation. The third section assessed knowledge and awareness of teledentistry, including familiarity with the term, prior experience, and understanding of the store-and-forward model. The final section evaluated attitudes and feasibility, covering perceived usefulness, anticipated frequency of use, acceptable response times, and the assessment of the University Hospital Aachen as a potential regional provider of teledentistry services.

### Data collection, processing and statistical analysis

Data collection took place between May and December 2023 through both online and paper-based formats. Data were analysed using SPSS (version 29.0.1.1 for Windows 11). Online survey responses were automatically formatted for SPSS analysis, while paper responses were manually entered into the SoSci Survey system for consistency. Frequency distributions were visualized using bar and pie charts. Contingency tables were employed to compare categorical variables, such as professional specialisation and years of experience, or digitalisation levels and radiographic techniques. To test for statistical significance, chi-square tests were applied. To apply the chi-square test, corresponding null hypotheses (H(0)) were formulated for the alternative hypotheses (H(1)) to be tested. When cell counts fell below the threshold for chi-square assumptions, Fisher-Freeman-Halton exact tests were used. Statistically significant findings were supplemented with Cramér’s V to assess effect size. The significance level for this study was set at *P* < .05. As this study was designed as an exploratory survey of the entire accessible regional dentist population, an a priori power calculation was not applicable.

### Declaration of generative AI and AI-assisted technologies in the writing process

During the preparation of this work the authors used Chat GPT and DeepL to improve readability and language. After using these tools, the authors reviewed and edited the content as needed and take full responsibility for the content of the published article.

## Results

### Demographic data and descriptive statistics

The present survey focused on the 575 private dentists and dental residents in the Aachen region. It was possible to reach out to a number of 362 dentists and dental specialists who were asked to participate in the study.

The cumulative response rate of online and paper surveys was 52% (*n* = 187). The majority (82%) were general dental practitioners, followed by orthodontists (10%), oral surgeons (3%), and oral and maxillofacial surgeons (3%); approximately 2% were residents in training. Most participants (79%) had more than ten years of professional experience.

### Practice structures among respondents

Slightly more than one-third of respondents worked independently in private practice, while another third collaborated with one other dentist. About 12.5% reported working in teams of three, and 17% in practices with three or more colleagues. Among larger practices, 28.5% consisted of four, seven, or fifteen dentists, and 14% reported working in five-dentist settings.

Across all specialties, both solo and multi-dentist practices were represented. Among general practitioners, 36% worked alone, 35% in pairs, and 29% in groups of three or more. Comparable patterns were found across specialties, although oral and maxillofacial surgeons mainly practiced in multi-dentist settings. Dental residents exclusively worked in multi-dentist environments, most commonly in teams of two or more. These findings provide structural context for subsequent analyses of attitudes toward store-and-forward teledentistry, as practice size was one of the examined variables.

### State of digitalisation in dental practices

**Fig. 1:**
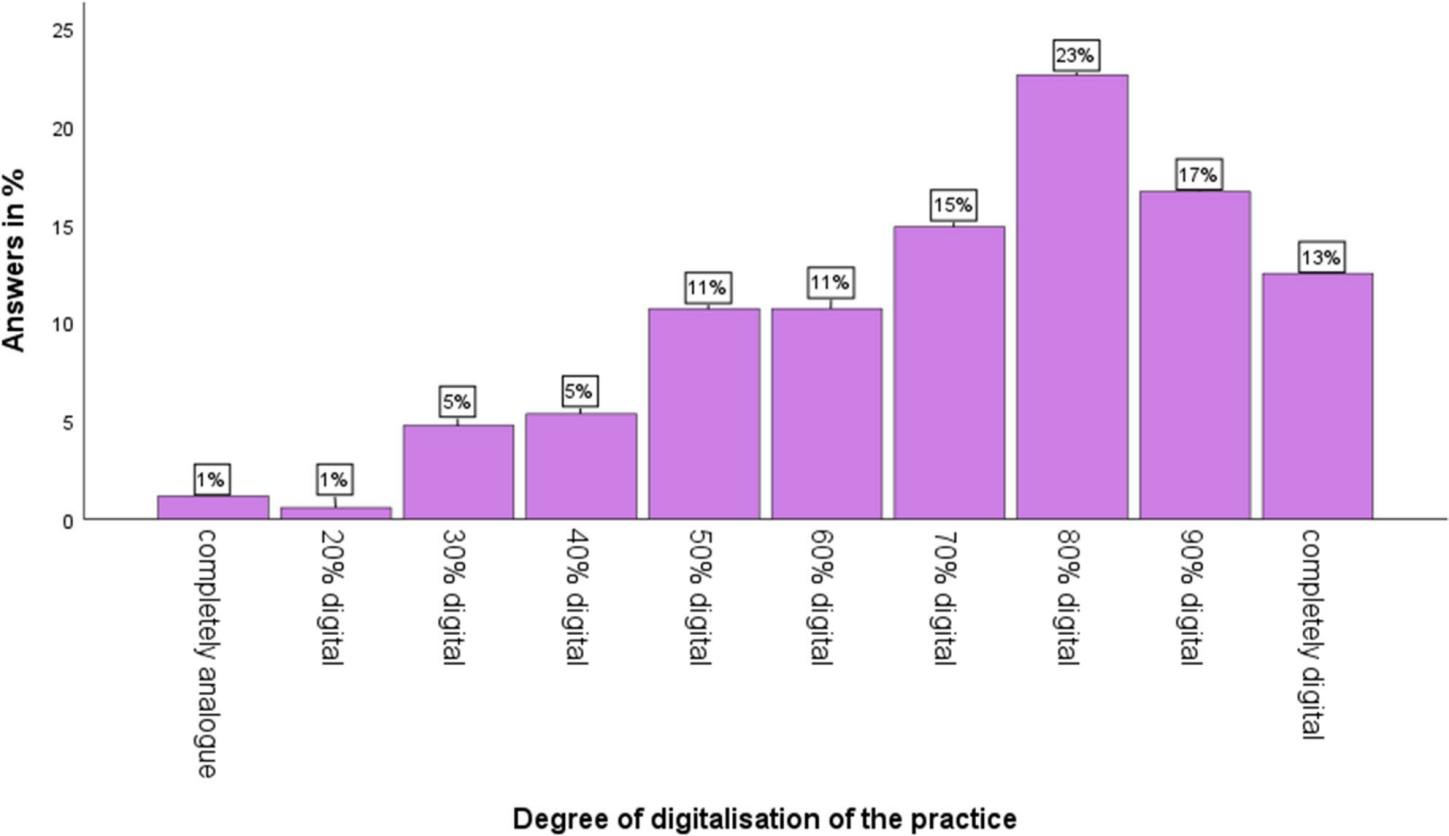
Degree of digitalization in dental practices within the Aachen region

The level of digitalisation in dental practices averaged between 70% and 80%. As shown in Fig. 1, nearly 2% of practices have integrated little to no digital technology into their daily workflow, whereas approximately 13% report being fully digitalised. A key component of digitalisation is the use of digital radiographic imaging. 84% of practices utilise digital radiographic systems or digitise analogue images using scanners. 16% still rely on analogue radiographic techniques without digital storage capabilities. The degree of digitalisation correlated significantly with the type of radiographic technology used. Although the association between digitalisation level and attitudes toward teledentistry was not formally tested, the descriptive data indicated that most participants already work in digitally equipped environments. Regarding internet quality, over 75% of respondents rated their connection as good or very good. Slightly more than 20% consider it adequate, while 2% describe it as poor. However, all surveyed practices have at least basic internet access.

### Teledentistry

#### Familiarity with teledentistry and response time expectations

**Fig. 2:**
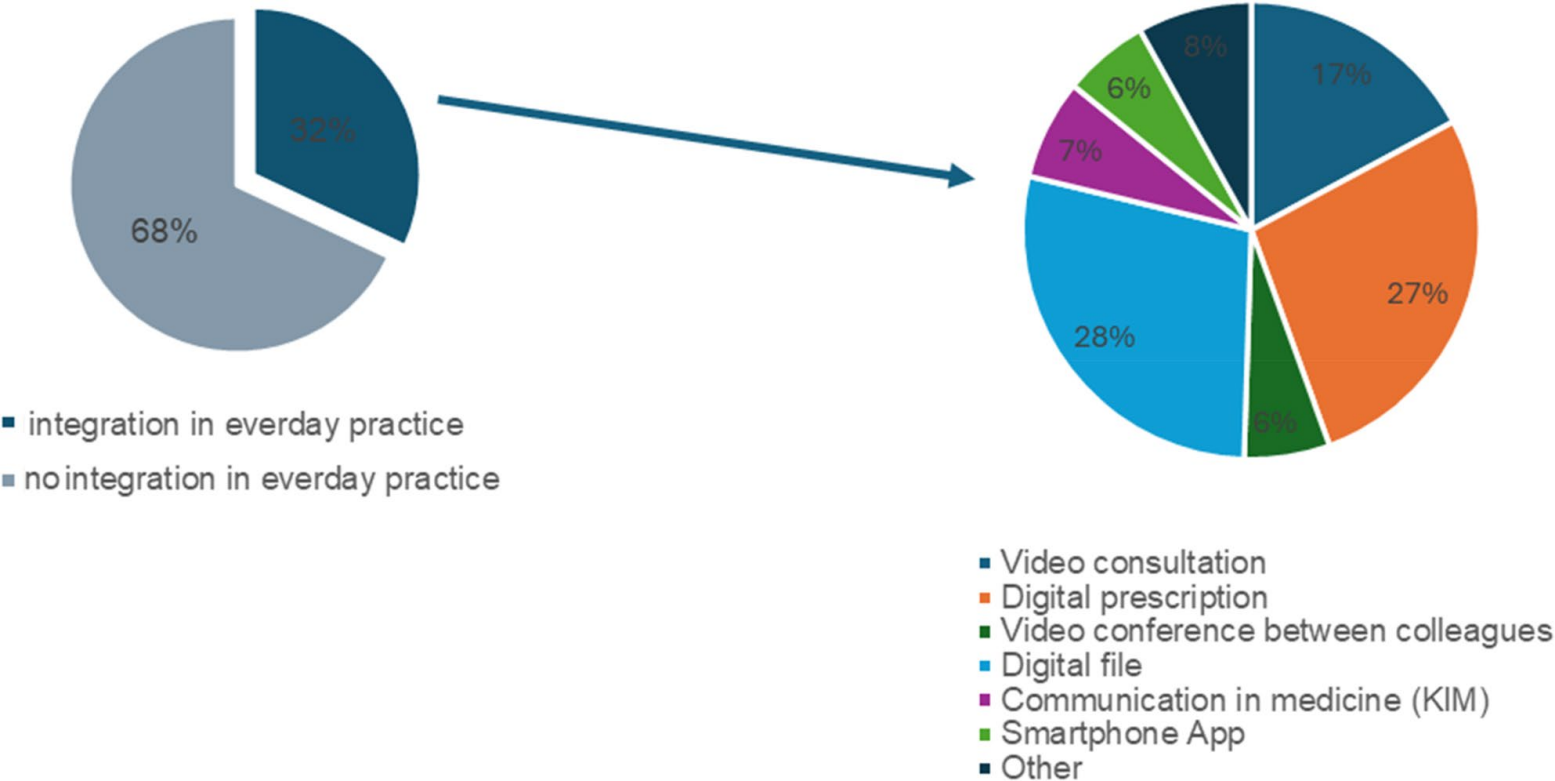
Percentage of teledental applications in dental practices

More than 70% of surveyed dentists reported being familiar with the term teledentistry; however, only 25% correctly identified its full scope. While remote diagnosis, professional video consultations, and online consultations were most commonly recognised as teledentistry services (48–60%), other components such as digital patient records, electronic prescriptions, online monitoring, and smartphone applications were less consistently associated with the concept (20–36%). Overall, 32% of practices had already implemented at least one teledental application, including video consultations, digital patient management, secure medical communication systems, or app-based and AI-supported tools (Fig. 2).

**Fig. 3:**
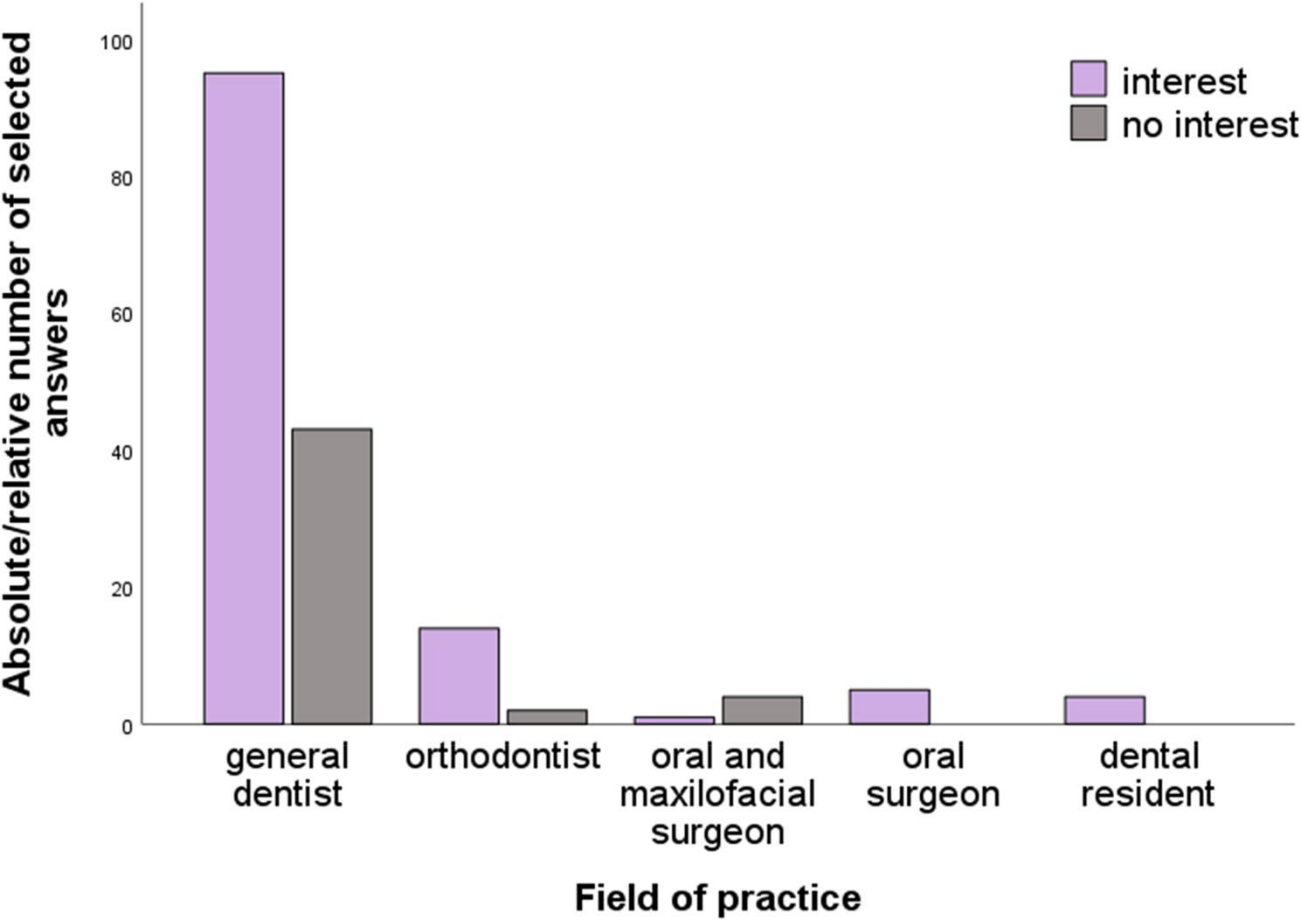
Interest in teledentistry services based on the store-and-forward model, divided by field of practice

A store-and-forward teledentistry concept where dentists can refer complex patient cases, is considered useful by 71% of respondents, while 29% do not see a need for such a concept. An examination of personal interests, broken down by area of specialisation, revealed that 100% of oral surgeons and 100% of residents support the idea of a teledentistry centre based on the store-and-forward model. Sixty-nine per cent of general dentists in private practice and 88% of orthodontists perceive a personal benefit from such a centre. Eighty per cent of oral and maxillofacial surgeons have no interest. (Fig. 3).

Regarding the appropriate timeframe for a specialist’s response, 47% of surveyed dentists considered a response time of two to three working days to be sufficient. Slightly more than 25% prefer a faster reply, stating that a maximum of one day is appropriate. Approximately 12.5% of dentists expect a response within half a working day. Meanwhile, 5% of respondents believe a response should be provided within one to two hours. Approximately 8% consider a timeframe of up to one week acceptable for receiving a specialist’s diagnosis and treatment recommendations. When analysing these response time preferences in relation to different areas of dental specialisation, no significant differences were observed.

### Concerns regarding the use of teledentistry services

**Fig. 4:**
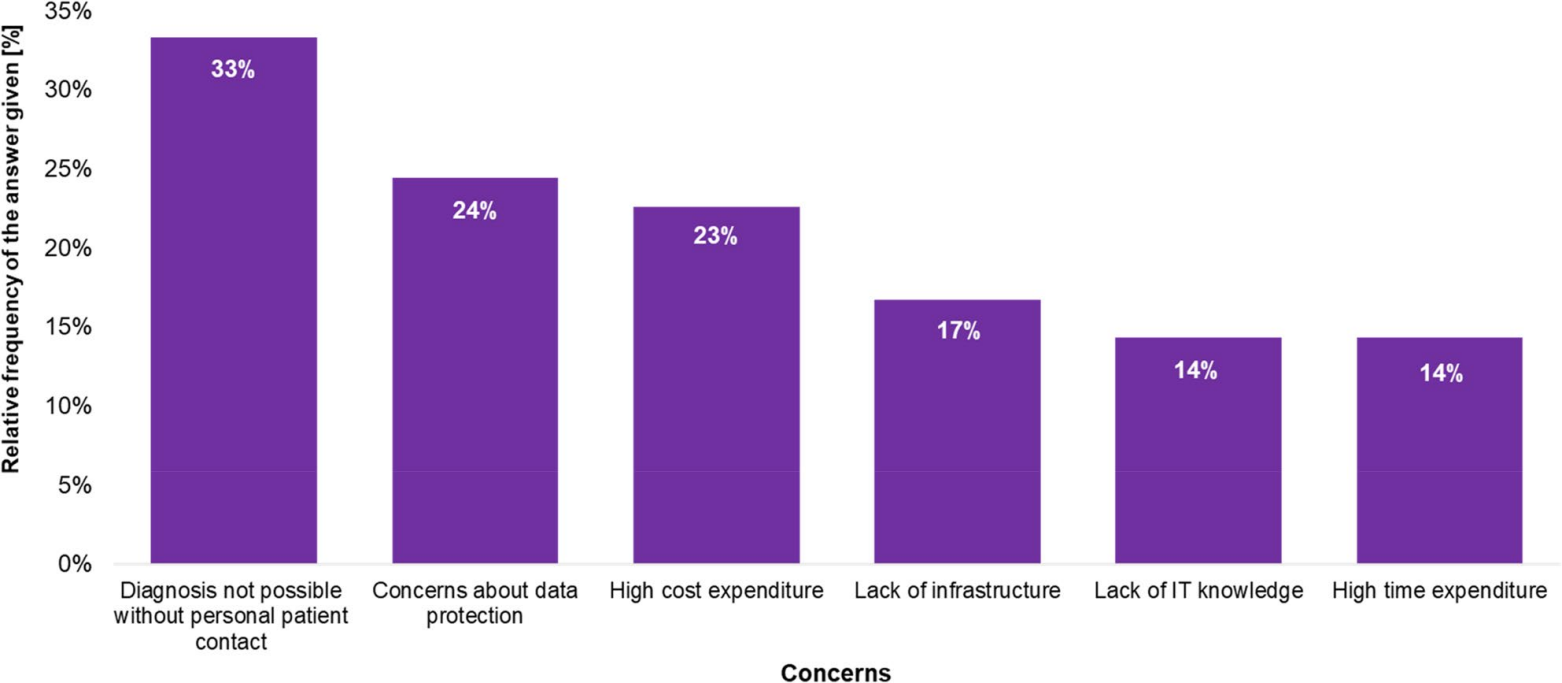
Ranking of applications of teledental consultations by the dentists

More than 50% of the surveyed dentists expressed no concerns about using teledentistry services. However, 43% remain sceptical about utilising digital communication for consultations with specialists. Of these, approximately 80% selected one to three concerns from the six listed response options. Nearly 4% expressed concerns regarding all potential issues. The most frequently mentioned concerns, in descending order, were inability to diagnose without direct patient contact, data protection concerns, high investment and operational costs, lack of infrastructure accounted for 17% of the concerns, increased time requirements compared to traditional methods, and insufficient IT knowledge (Fig. 4).

### Potential applications for teledentistry consultations

**Fig. 5:**
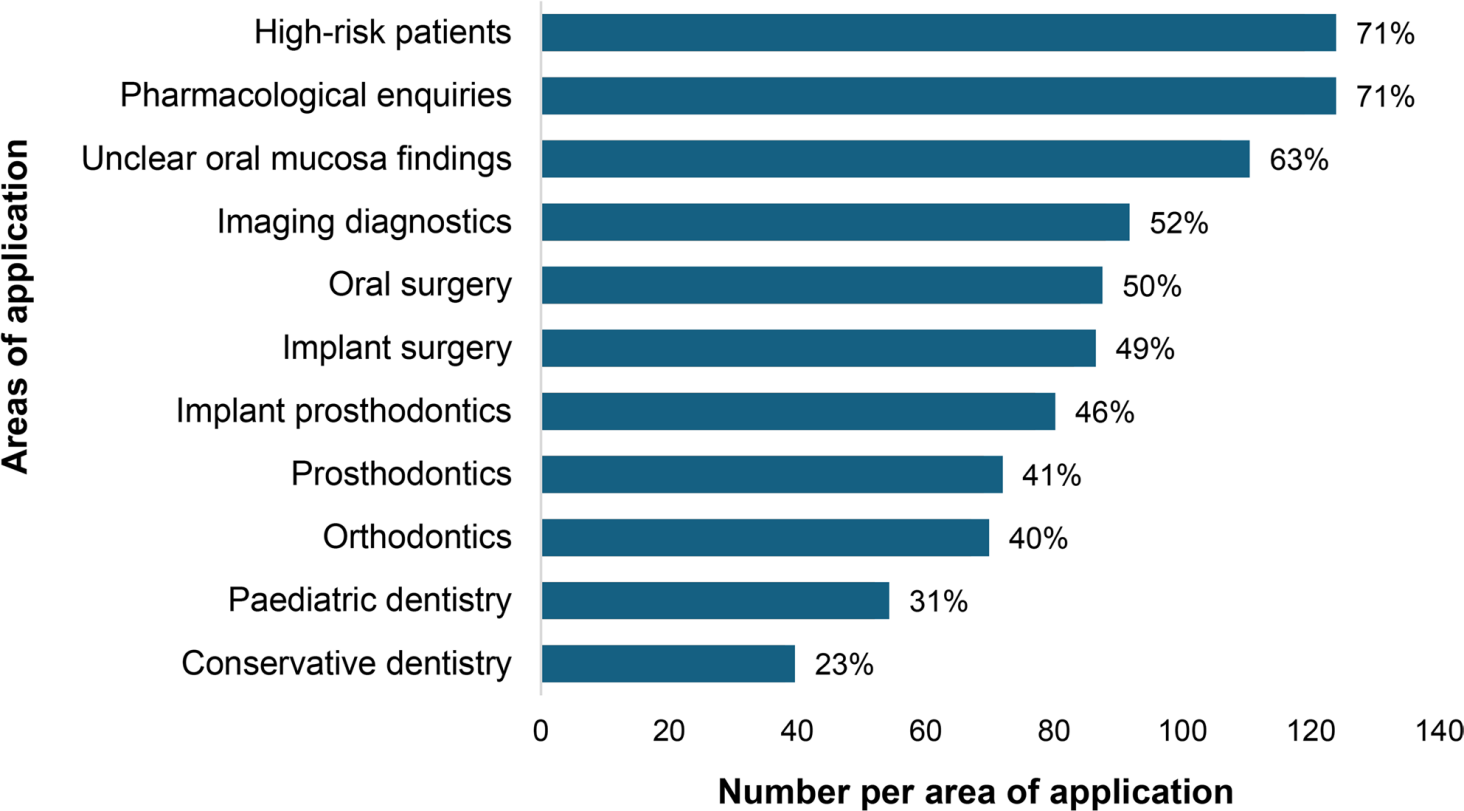
Relative frequency of concerns toward teledental applications

Respondents were allowed to select multiple answers, and on average, dentists chose five out of eleven possible application areas. Notably, approximately 13% of respondents indicated that they would use teledentistry services for only one specific application, while another 13% expressed willingness to utilise these services across all eleven listed areas. A ranking of the responses is illustrated in Fig. 5. Treatment of high-risk patients and pharmacological inquiries regarding drug intolerances and interactions were both selected in 71% of responses, followed by unclear oral mucosal lesions, which ranked second with 63% of respondents stating they would seek teledentistry advice for this issue. Consultation for radiographic diagnostics and teledentistry support in oral and maxillofacial surgery were each selected in 50% of responses. Implant surgery, implant prosthetics, and general prosthetic inquiries were also recognized as relevant applications, ranking slightly below the previous categories. Orthodontic cases were identified as a potential application in nearly 40% of responses, and conservative dentistry inquiries ranked lowest among the potential use cases.

### Personal interest in teledentistry services based on the store-and forward model in dependence on different factors

#### Hypothesis 1: years of professional experience

It was tested if the interest in teledentistry services (store-and- forward concept) is dependent on the years of professional experience. Within the studied sample, no significant correlation was found (*P*=.76, Fisher-Freeman-Halton exact test). Overall, a higher proportion of dentists expressed interest in teledentistry services based on the store-and-forward model than those who did not, but this interest was independent of their years of practice. The null hypothesis was accepted.

#### Hypothesis 2: potential frequency of use

A correlation between personal interest and the potential frequency of use of teledentistry services based on the store-and-forward model was also examined. The contingency table (Table 1) shown here already indicates a potential relationship and highlights a significant discrepancy between expected and actual responses. Dentists with fewer than one relevant case per quarter perceived less personal benefit from teledentistry services based on the store-and-forward model than expected, whereas those with one to three such cases per quarter reported a considerably higher perceived benefit (+ 11%). For those with four to ten relevant cases per quarter, interest was even 20% higher than expected.

To test the hypothesis, dentists were divided into two groups: 1. those who perceived a benefit from teledentistry services based on the store-and-forward model and 2. those who saw no personal benefit. When comparing these two groups with the reported frequency of cases in which they would seek teledentistry consultation, a significant difference of *P*<.01 with a large effect size (Cramér’s V = 0.449) was obtained. Within the studied sample, a significant correlation existed between the perceived personal benefit of teledentistry services based on the store-and-forward model and the reported frequency of cases in which dentists would use such a service. The null hypothesis was rejected.

**Table 1:** Contingency table of personal benefit of teledentistry services based on the store-and-forward model in correlation with the number of expected treatment cases per quarter

			Personal benefit of teledentistry services based on the store-and-forward concept	
			beneficial	not beneficial
**Number of treatment cases** **per quarter**	< 1	Number	17	28
		Expected number	32	13
	1–3	Number	52	12
		Expected number	45	19
	4–10	Number	29	3
		Expected number	23	9
	> 11	Number	21	6
		Expected number	19	8

#### Hypothesis 3: number of dentists in a practice

Finally, it was tested whether there is a correlation between the potential use of teledental consultations and the number of dentists working in a practice. The chi-square test for independence yielded an asymptotic significance of *P*=.598. Consequently, the null hypothesis was accepted, indicating no statistically significant correlation between the number of dentists in a practice and a decreased interest in teledentistry consultations.

## Discussion

The present study examined dentists’ awareness, knowledge, and acceptance of teledentistry services in general, and of the store-and-forward model in particular and explored their interest in a regional teledentistry centre. The data obtained provide valuable insights into the attitudes of the target group, as well as the infrastructural conditions relevant to the potential implementation of such a centre.

Many respondents were experienced general practitioners, most of them with over ten years of professional experience, reflecting the demographic composition of the regional dental workforce. Similar findings were reported by Löhrs-Hintz in her study on dentists’ knowledge of teledentistry in Brandenburg, Germany [22].

Most respondents believed they were familiar with the term “teledentistry”. This overall positive result in terms of knowledge is consistent with a previous study reporting that 68.6% of dentists considered themselves familiar with teledentistry [30]. However, evidence in the literature is not entirely consistent. In a recent national survey from France, Giraudeau et al. [29] found that 57% of private dentists had never heard of teledentistry, highlighting substantial variability in awareness across countries and professional environments. Such discrepancies may reflect differences in digital infrastructure, exposure to telehealth initiatives, and the degree to which teledentistry has been integrated into national dental education. Interestingly, only 25% of the respondents in this study were able to correctly categorise the components of teledentistry. The contrast between perceived familiarity and actual understanding indicates that, although teledentistry is viewed as relevant, its underlying mechanisms remain insufficiently understood in routine practice. Educational gaps have been highlighted in different studies calling for structured teledentistry training [33–35].

Consistent with recent studies highlighting growing professional acceptance of asynchronous consultation models [14], respondents expressed broad interest in store-and-forward consultations for complex or interdisciplinary cases. The majority recognised the potential usefulness of establishing a regional teledentistry centre to support such interactions. Overall, 71% of participants regarded the store-and-forward teledentistry model as beneficial. Comparable international studies have similarly reported that the advantages of teledentistry generally outweigh its disadvantages [25, 26, 30, 36, 37]. The most frequently identified application areas included consultations for high-risk patients, pharmacological inquiries, and unclear oral mucosal lesions. These scenarios underscore the perceived value of teledentistry, particularly when a prompt expert opinion is needed to clarify complex conditions or to manage interdisciplinary cases. Prior research confirms the diagnostic reliability of such remote assessments: Queyroux et al. [38] demonstrated a high degree of agreement between video-based and face-to-face diagnoses, with reported sensitivity and specificity of 94%, and other studies have shown similar effectiveness in detecting oral lesions [39].

The findings on technical infrastructure are encouraging and suggest that the prerequisites for implementing teledentistry are already largely in place. More than 80% of participating practices reported using digital radiography, and overall digitalization levels between 70% and 80% indicate substantial readiness for digital communication. Internet connectivity was also rated as good to very good in most cases. Only a minority of respondents expressed doubts about the adequacy of their technical equipment, which points to increasing integration of digital tools into daily workflows. The expectations regarding response times further support the feasibility of asynchronous, store-and-forward teledentistry. Most dentists considered a turnaround of two to three working days sufficient, suggesting that remote consultation can realistically complement routine clinical practice without time pressure or workflow disruption. These pragmatic expectations are consistent with previous reports highlighting the compatibility of asynchronous models with everyday dental care [16, 40]. Even though studies have shown that remote diagnostics are effective in various dental fields, 43% of the surveyed dentists were sceptical about digital services, particularly regarding diagnoses without direct patient contact. Another concern was the secure transmission of patient data. Data privacy using teledental applications seems to be a global concern [25, 27, 41].

Cost concerns were raised by 23% of dentists, yet the existing fee scale for dentists in Germany allow teledental and video consultations to be reimbursed [42]. However, the current reimbursement rate may not adequately reflect the time required for effective consultation. From a policy and educational perspective, our findings highlight the need for clearer regulatory guidance, adequate reimbursement frameworks, and structured training opportunities.

As acceptance of teledentistry in this study was largely driven by knowledge and familiarity, structured training modules should be integrated into both undergraduate and continuing dental education. Evidence suggests that targeted educational interventions can positively influence attitudes toward teledentistry and increase confidence in its clinical application [34]. Such training may include case-based learning, hands-on exposure to store-and-forward workflows, and interdisciplinary consultation exercises that specifically address data protection requirements and diagnostic limitations. Moreover, digital and tele-education approaches in dental curricula have been shown to improve learning outcomes, clinical skill development, and professional confidence with information and communication technologies, supporting their suitability for preparing dentists to effectively adopt teledentistry in routine practice [43, 44]. Recent implementation studies further emphasize the importance of clearly defined learning objectives, delivery methods, and assessment strategies to ensure sustainable and effective teledentistry training [45].

Contrary to initial assumptions, the study found no correlation between interest in teledentistry and years of professional experience (*P*=.76). This suggests that teledentistry is perceived as potentially valuable across all experience levels, indicating broad relevance and appeal. However, findings by Brüllmann et al. [46] suggest that younger dentists could benefit from the expertise of senior colleagues via teledental consultations, and also Löhrs-Hintz [22] found that younger dentists were more interested in teledentistry than experienced practitioners. It must also be considered at this point that most respondents had more than ten years of professional experience, with fewer respondents from younger or less experienced dental professionals. As a result, the findings might be skewed toward the perspectives of more experienced dentists, whose views on teledentistry might differ from those of younger professionals or dental trainees. Moosa et al. [47] found in their survey that dentists with less than five years of professional experience expressed particularly strong concerns regarding data security.

The second hypothesis was clearly supported. There was a statistically significant correlation between the frequency of potential application scenarios and the perceived benefit of a teledentistry based on the store-and-forward concept (*P* < .01). Dentists who anticipated a higher number of relevant cases per quarter were more likely to see value in using such a service. This finding underscores a strong practical orientation and case-based approach among the respondents. Specialisation also influences interest: oral surgeons and dental residents showed greater interest, whereas 80% of maxillofacial surgeons opposed a teledentistry center. These findings differ from those of Löhrs-Hintz [22], potentially due to the small sample size. Other studies [27, 36] have not found significant differences across specializations.

The third hypothesis - that larger practices with more dentists would be less inclined to use teledentistry - was not supported. The number of dentists employed in a practice had no significant impact on acceptance (*P*=.598). This indicates that the decision to engage with teledentistry may be more influenced by individual needs and workflows than by practice size.

This study was limited to a region in Germany and may not be directly generalizable to other areas as the study sample may not be fully representative of the entire dental community. Aachen as a location with a university hospital may offer easier access to academic resources, professional networks, and digital infrastructure. This proximity could influence both the openness to digital technologies and the level of awareness regarding teledentistry. In contrast, dental practices in rural or structurally underserved areas may face greater challenges, such as limited technical infrastructure and reduced access to continuing education. Future studies should therefore focus on these regions, where the need for teledental services may be even more pronounced due to geographic and logistical constraints.

Furthermore, a self-selection bias cannot be ruled out, as dentists with a greater interest in digital tools may have been more inclined to participate. This could affect the representativeness of the findings, particularly concerning perceived barriers and concerns. Lastly, due to the cross-sectional design, the study provides only a snapshot of current attitudes and practices. Longitudinal research is needed to assess how perceptions of teledentistry evolve over time as experience, infrastructure, and policy frameworks change.

## Conclusions

This study highlights the promising potential of store-and-forward teledentistry services, particularly for dentists who frequently manage specialized or complex treatment cases. The concept was well received among participants, indicating openness toward integrating digital consultations into routine practice. While years of professional experience did not significantly affect interest, dentists with higher case frequencies were more likely to recognize the benefits of teledentistry. However, concerns about data protection and the inability to diagnose without direct patient contact remain barriers that need to be addressed. Future research should focus on addressing these concerns, adjusting reimbursement models, ensuring adequate infrastructure and developing potential solutions to promote the integration of teledentistry into daily practice. Integrating teledentistry into education and training programs could enhance overall knowledge levels.

## Supplementary Information

Supplementary Material 1.

## Data Availability

The datasets used and analysed during the current study are available from the corresponding author on reasonable request.

## Footnotes

1 TELnet@NRW: telemedical, cross-sectoral network as a new digital structure to measurably improve local healthcare delivery (a project to establish a telemedical network in Northrhine Westphalia, Germany).
