# Supplementary material for: Awareness, knowledge, and acceptance of teledentistry among dental practitioners: a cross-sectional survey
Source: BMC Oral Health. 2026 Feb 24;26:554. doi: 10.1186/s12903-026-07931-5 (PMC13037253; doi:10.1186/s12903-026-07931-5)
Supplement: Supplementary file 1 — Supplementary Material 1. [file 12903_2026_7931_MOESM1_ESM.pdf]

UNIKLINIK RWTHAACHEN
Klinik für Zahnärztliche Prothetik und Biomaterialien · Zentrum für Implantologie

Klinikdirektor
Univ.-Prof. Dr. med. dent.
Stefan Wolfart

Survey on Teledentistry

Dear Colleague,
We kindly ask you to answer 16 questions on the topic of teledentistry. The survey will take approximately 5 minutes. Please indicate which statement(s) best apply to you.

For improved readability, gender-inclusive language is not used in this questionnaire. However, all references to persons apply equally to all genders.

All data will be collected anonymously, analyzed in strict confidence, and used solely for the purposes of this study.

By completing and returning the questionnaire, you agree to the use of your responses for research purposes.

Thank you very much for your participation!

1. **I work as a:**
   ☐ Dentist
   ☐ Specialist in Orthodontics
   ☐ Specialist in Oral and Maxillofacial Surgery
   ☐ Specialist in Oral Surgery
   ☐ Dental Resident

2. **How long have you been practicing?**
   ☐ < 2 years
   ☐ 2–5 years
   ☐ 5–10 years
   ☐ > 10 years

3. **How many dentists work in your practice?**
   ☐ 1
   ☐ 2
   ☐ 3
   ☐ > 3, please specify: ______

4. **How would you rate the level of digitalization in your practice?**

	1	2	3	4	5	6	7	8	9	10	
analog	☐	☐	☐	☐	☐	☐	☐	☐	☐	☐	fully digital

5. **How would you rate your practice's internet connection?**
   ☐ Excellent
   ☐ Good

☐ Adequate
☐ Poor
☐ No internet access

6. **Do you work with digital X-rays?**
   ☐ Yes, analog images are scanned
   ☐ Yes, we use digital imaging
   ☐ No
7. **Are you familiar with the term *teledentistry*?**
   ☐ Yes
   ☐ No
8. **Which of the following do you associate with teledentistry? (Multiple answers possible)**
   ☐ Smartphone apps
   ☐ Digital prescriptions
   ☐ Electronic health records
   ☐ Diagnosis and consultation at a distance
   ☐ Online/telephone consultations
   ☐ Online patient monitoring
   ☐ Video consultations between colleagues
   ☐ All of the above belong to teledentistry
9. **Have you already implemented any form of teledentistry in your practice?**
   ☐ Yes, if so, which: ___________________________
   ☐ No

The following section refers to a specific use case of teledentistry: A general dentist submits a digital consultation request to a specialized colleague. This request includes an electronic patient record with clinical data, radiographs, and, for example, photographs. Based on this material, the specialist provides a diagnosis and therapeutic recommendation, which is shared digitally and/or discussed via video consultation.

10. **Do you believe this model of teledentistry could be helpful for you personally?**
    ☐ Yes
    ☐ No, because ____
11. **What turnaround time for the specialist's response would you consider appropriate?**
    ☐ 1–2 hours
    ☐ Half a working day
    ☐ 1 working day
    ☐ 2–3 working days
    ☐ 1 working week

12. **Do you have any fundamental concerns about using telemedicine services?**
    ☐ No
    ☐ Yes

13. **If you answered “Yes” in question 12: What are your concerns regarding teledentistry? (Multiple answers possible)**
    ☐ Data protection/privacy
    ☐ Diagnosis is not possible without direct patient contact
    ☐ More time-consuming than conventional methods
    ☐ High cost (investment/operational)
    ☐ Lack of IT skills
    ☐ Inadequate infrastructure

14. **For which types of cases do you see potential for teledentistry? (Multiple answers possible)**
    ☐ Prosthetic issues
    ☐ Implant-prosthetic issues
    ☐ Implant-surgical issues
    ☐ Orthodontic issues
    ☐ Maxillofacial surgical issues
    ☐ Unclear mucosal lesions
    ☐ Pharmacological queries (e.g. drug interactions)
    ☐ Issues related to treatment of high-risk patients
    ☐ Pediatric dentistry
    ☐ Imaging consultation
    ☐ None, because ____

15. **How frequently do you encounter cases (on average per quarter) where you would seek teledentistry advice?**
    ☐ < 1
    ☐ 1–3
    ☐ 4–10
    ☐ > 11

16. **Do you consider the University Hospital RWTH Aachen and its dental departments suitable as a regional center for teledentistry?**
    ☐ Yes
    ☐ No, because ____________________________
